# Rhynchophylline Regulates Calcium Homeostasis by Antagonizing Ryanodine Receptor 2 Phosphorylation to Improve Diabetic Cardiomyopathy

**DOI:** 10.3389/fphar.2022.882198

**Published:** 2022-04-19

**Authors:** Jiao Liu, Yating Zhao, Yufang Zhu, Yan Wang, Xiaoshuang Liu, Xiaobo Nie, Jing Zhao, Wei Wang, Jie Cheng

**Affiliations:** ^1^ College of Basic Medicine, Hebei Medical University, Shijiazhuang, China; ^2^ College of Pharmacy, Hebei University of Chinese Medicine, Shijiazhuang, China; ^3^ College of Integrated Chinese and Western Medicine, Hebei Medical University, Shijiazhuang, China

**Keywords:** rhynchophylline, type 2 diabetes mellitus, myocardial lesions, calcium homeostasis, mitochondria

## Abstract

Diabetic cardiomyopathy (DCM) is a serious complication of diabetes that can lead to heart failure and death, for which there is no effective treatment. Rhynchophylline (Rhy) is the main effective component of the Chinese herbal medicine *Uncaria rhynchophylla*, which mainly acts on the cardiovascular and nervous systems. However, its role in protecting against DCM remains unexplored. The present study sought to reveal the mechanism of Rhy in improving type 2 diabetes mellitus (T2DM) myocardial lesions from the perspective of regulating calcium homeostasis in cardiomyocytes. We prepared a mouse model of T2DM using a high-fat diet combined with low doses of streptozotocin. The T2DM mice were given 40 mg/kg of Rhy for 8 weeks. The results showed that Rhy can attenuate cardiac pathological changes, slow down the heart rate, decrease serum cardiac enzyme levels, reduce cardiomyocyte apoptosis, enhance cardiomyocyte contractility, and raise the calcium transient amplitude in T2DM mice. Further, Rhy downregulated the phosphorylation level of ryanodine receptor 2, upregulated the phosphorylation level of phospholamban, protected mitochondrial structure and function, and increased adenosine triphosphate levels in the cardiac tissue of T2DM mice. Our results demonstrated that Rhy may protect against myocardial damage in T2DM mice and promote cardiomyocyte contraction, and its mechanism of action seems to be related to the regulation of intracellular calcium homeostasis.

## Introduction

Nowadays, diabetes has become a global problem threatening human health. With the increasing incidence of diabetes, various complications have also triggered widespread concern. Diabetic cardiomyopathy is considered to be a disorder of cardiac structure and function independent of hypertension, coronary heart disease, and related heart diseases (Boudina & Abel, 2007). It has been reported that the main characteristics of diabetic cardiomyopathy are myocardial dilatation or hypertrophy and left ventricular (LV) systolic or diastolic dysfunction (Devereux et al., 2000). There is currently a lack of exclusive drugs for the treatment of DCM in clinical practice. Therefore, it is important to search for potential compounds with protective effects against DCM.

The excitation–contraction coupling of cardiomyocytes is related to the change in Ca^2+^ concentration in cytoplasm (Greenstein & Winslow, 2011). Between 10 and 20% of intracellular Ca^2+^ flows in through L-shaped calcium channels, and 80–90% is released from the sarcoplasmic reticulum. Ca^2+^ is released from the sarcoplasmic reticulum to the cytoplasm through ryanodine receptor 2 (RyR2), then returned to the sarcoplasmic reticulum through sarco-endoplasmic reticulum Ca^2+^ adenosine triphosphate (ATP)ase (SERCA) by ATP consumption (James et al., 1989; Boardman et al., 2014). RyR2 and SERCA cooperate closely to maintain the dynamic balance of the sarcoplasmic reticulum calcium reserve. A decrease in SERCA activity will directly lead to a decrease in the calcium recovery capacity of the sarcoplasmic reticulum, resulting in a reduced calcium reserve of the sarcoplasmic reticulum and the destruction of normal calcium homeostasis of cardiomyocytes (Eisner et al., 2020). It has been reported that diabetes can cause calcium balance changes and abnormal calcium homeostasis in cardiomyocytes. With the serious loss of Ca^2+^ in the sarcoplasmic reticulum of cardiomyocytes, the calcium reserve in the sarcoplasmic reticulum and the myocardial contractility decrease (Ly et al., 2017; Durak et al., 2022).

Ca^2+^ in mitochondria is also an important regulator of the intracellular calcium dynamic balance. Mitochondria have a perfect Ca^2+^ uptake and release system, which can quickly sense changes in intracellular Ca^2+^ concentrations, thereby regulating the respiratory function of mitochondria (Dia et al., 2020). Mitochondria are regulators of intracellular Ca^2+^ (Miranda-Silva et al., 2020). With an increased Ca^2+^ concentration in cytoplasm, the uptake of Ca^2+^ by mitochondria also increases to restore the concentration in the cytoplasm, but the recovery of Ca^2+^ concentration in the mitochondrial matrix is slower than that in cytoplasm (Suarez et al., 2018). Scientists once thought that an increase in Ca^2+^ concentration in cytoplasm was the main cause of cell damage (Njegic et al., 2020), but the latest research suggests that the root cause of cell death is actually mitochondrial calcium overload (Pinton et al., 2008). Studies have confirmed that mitochondrial calcium accumulation exists in common senile diseases, such as neurodegenerative diseases (Liao et al., 2017), type 2 diabetes (Tuncay et al., 2017), and muscle atrophy (Su et al., 2021). Reactive Oxygen Species (ROS) production increases during the progression of T2DM. The increase in ROS serves to decrease the antioxidant capacity of the diabetic myocardium, contributing significantly to oxidative stress and resultant myocardial mitochondrial damage (Wold et al., 2005). Disruption of mitochondrial structure, which increases Ca^2+^ inward flow, also contributes to mitochondrial calcium overload.

**FIGURE 1:**
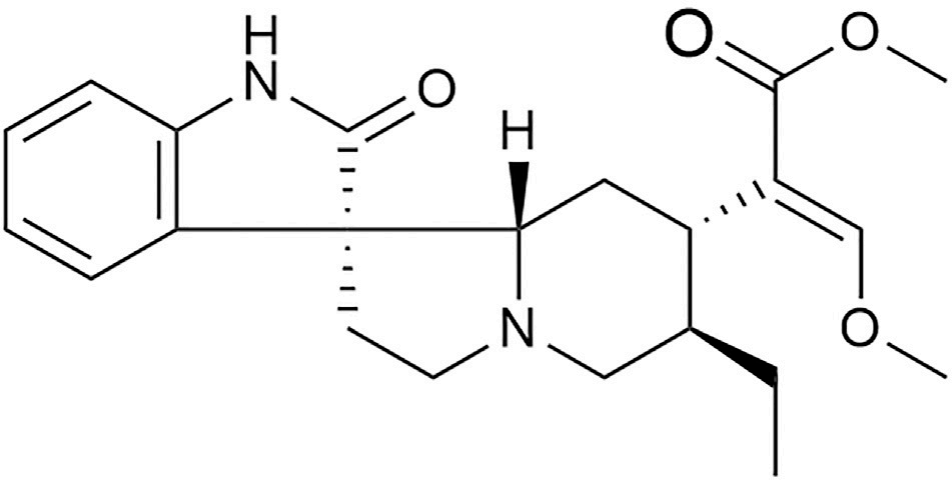
Chemical structure of rhynchophylline.

Rhynchophylline (Rhy) (chemical structure, Figure 1) is the main effective component of the Chinese herbal medicine *Uncaria rhynchophylla*, which has good effects on the cardiovascular system (Zhou & Zhou, 2010). Previous studies have demonstrated that Rhy may have cerebral ischemia–protection, calcium channel–blocking, myocardial remodeling–inhibition, and anti-arrhythmia effects (Qin et al., 2019). However, little is known regarding the role of Rhy in DCM.

In view of the fact that inhibition of cytoplasmic calcium overload protects cardiomyocytes, we speculated that Rhy, a calcium antagonist, may regulate intracellular calcium homeostasis and have a beneficial therapeutic effect in DCM. Therefore, in this manuscript, we explored the mechanism of Rhy in improving T2DM myocardial lesions from the perspective of regulating the release and recovery of sarcoplasmic reticulum calcium ions and mitochondrial function.

## Materials and Methods

### Drugs and Chemicals

Rhy (76-66-4) was purchased from Shanghai Yuanye Biotechnology Co., Ltd. (Shanghai, China). Dantrolene (DTL) (D9175) and streptozotocin (STZ) were purchased from Sigma-Aldrich (St. Louis, MO, United States). Purity levels were all > 98%.

### Animals and Experimental Protocols

The animals used in our experiments were provided by the Hebei Experimental Animal Center. C57BL/6 healthy mice aged 12–14 weeks were selected and fed freely and given water in a specific pathogen-free environment with the ambient temperature ranging from 23 to 25°C. We treated all experimental animals humanely and followed the Guidelines for the Management and Use of Experimental Animals issued by the U.S. National Institutes of Health. The experimental procedures were approved by the Experimental Animal Ethics Committee of Hebei University of Chinese Medicine.

The C57BL/6 mice were adaptively reared for 1 week. After fasting them for 12 h, we recorded their body weight and blood sugar values, then selected those mice with normal blood sugar and moderate body weight values as experimental mice.

After the mice were fed a high-fat diet for 4 weeks, they were intraperitoneally injected with STZ (70 mg/kg body weight) daily for five consecutive days. One week after the administration of STZ, those mice with a blood glucose concentration of > 10 mmol/L were considered diabetic. The feed used in the experiment was made and supplied by Beijing Keao Cooperative Feed Co., Ltd. (Beijing, China) according to regulations. The high-fat feed was the feed of the control group plus the addition of 30% lard to the feed.

The successfully modeled mice were randomly divided into three groups according to the balance of blood glucose level—namely, a type 2 diabetes mellitus (T2DM) group, a positive drug group (intragastric administration of 5 mg/kg of body weight daily of DTL), and a Rhy group (intragastric administration of 40 mg/kg of body weight daily of Rhy), with 10 mice in each group. Each group was given medicine once a day for 8 weeks. The control group (*n* = 10) was fed a normal diet and treated with 0.1 mol/L of citrate buffer.

### Blood Glucose Measurement

We inserted the blood glucose test paper into a blood glucose meter (Roche Holdings AG, Basel, Switzerland), cut off the tip of each mouse’s tail, squeezed from the tail root to the tail tip, dropped the blood that emerged from the tip onto the test paper, and read the results.

### M-Mode Echocardiography in Small Animals to Detect Left Ventricular Function

The mice were anesthetized with isoflurane, their hair from the xiphoid process to the neck was removed, and fixed on the ultrasonic heating table. The couplant for ultrasound was applied to the skin of the corresponding body surface over the heart, and a high-resolution small-animal imaging system (Vevo 770; FUJIFILM VisualSonics, Toronto, ON, Canada) was used to collect B-type ultrasound images of the parasternal LV short-axis section. The LV ejection fraction (EF, %), fraction shortening (FS, %), LV end-systolic volume (LVESV), and LV end-diastolic volume (LVEDV) were measured to evaluate the LV pumping function and systolic function. Meanwhile, the LV posterior wall thickness at end-diastole (LVPWd, mm), LV posterior wall thickness at end-systole (LVPWs, mm), interventricular septal thickness at end-diastole (IVSd, mm), and interventricular septal thickness at end-diastole (IVSs, mm) were measured to evaluate the ventricular myocardial thickness.

### Histopathological Analysis

Heart tissues were excised and rinsed in fixing fluid or liquid nitrogen for further testing. After ≥ 48 h, the heart tissues were removed from the fixing fluid, embedded in paraffin, stained with hematoxylin and eosin and Masson’s trichrome stain by a series of normative operations, then observed with a DM4000B microscope (Leica Microsystems, Wetzlar, Germany). Heart tissue sections were observed at a 400× field of view, and 5–10 fields were selected for pathology scoring. The analysis of collagen fiber area percentage in Masson’s trichrome–stained sections was performed using the ImageJ software (U.S. National Institutes of Health, Bethesda, MD, United States).

### Cardiac Injury–Associated Enzyme Measurement

Serum was separated from whole blood by centrifugation (1,301 g for 10 min) and used to measure creatine kinase (CK), lactate dehydrogenase (LDH), and aspartate aminotransferase (AST) levels according to the manufacturer’s instructions. Absorbances were measured using a UNICO-UV2000 spectrophotometer (JianCheng, Nanjing, China). The CK, LDH, and AST levels in mouse serum were measured with an assay kit (Biosino Bio-technology & Science Co., Ltd., Beijing, China).

### Acute Isolated Mouse Left Ventricular Cardiomyocytes

The mice were fixed and anesthetized, and thoracotomy was carried out. Hearts with a segment of aorta were quickly taken out and placed into calcium-free Tyrode’s solution. Under a microscope, the tissue around the heart was stripped clean and cut off from the first branch of the aorta. Each heart was quickly hung on a Langendorff perfusion device, and retrograde perfusion was performed via the aorta with 0.4-g/L type II collagenase-free Tyrode’s solution at 37°C for about 30 min. When the heart became soft and relaxed, the perfusion was deemed finished, the heart was removed, and the residual digestive juice was perfused and rinsed with 5 ml of KB solution. The LV was then shredded in KB solution and placed into the centrifuge tube. The cells were repeatedly blown into single cells by a suction tube. After filtration with a 200-mesh sieve, myocardial cells were obtained by precipitation.

### Measurement of the Contractility of Mouse Myocardial Cells

The Ion Optix single-cell edge-detection system (Ionoptix, LLC, Westwood, MA, United States) was used to detect cell-contraction function. Cells were incubated with 1.8 mmol/L of Ca^2+^ Tyrode’s solution for 10 min. An appropriate number of cells were placed in the bath and stimulated with an electric field of 10 V, 10 ms, and 1 Hz to find cells with clear edges and good shrinkage. Computer imaging was used to detect the edges of cells in real time, and their shrinkage and relaxation functions were measured. The shrinkage function of cells was evaluated for a duration of 1,000 s, focusing on shrinkage amplitude, peak time, and maximum shrinkage rate.

### Measurement of Calcium Transience in Mouse Cardiomyocytes

Dynamic changes in calcium transience in mouse LV myocardial cells were observed with calcium imaging technology. F-127 (Nalgene; Thermo Fisher Technology Co., Ltd., United States) and Fluo-4 AM (Sigma-Aldrich, St. Louis, MO, United States) were mixed evenly at room temperature, Tyrode’s solution containing 1.8 mmol/L of Ca^2+^ was added, and the mouse LV myocardial cell suspension was added for incubation for 40 min. A proper amount of cell suspension was absorbed and placed under an inverted fluorescence microscope (Leica Microsystems, Wetzlar, Germany), then stimulated with an electric field of 10 V, 10 ms, and 1 Hz. The myocardial cells with stable calcium transience for > 2 min were selected for recording, and then the dynamic parameters of calcium transience—namely, calcium transient amplitude and calcium recovery rate—were analyzed by software.

### Electron Microscopic Observation

The myocardial tissue (1 × 1 × 1 mm^3^) was quickly fixed in 2.5% pentanediol and 1% osmic acid, then dehydrated with ethanol of gradient concentrations and 100% acetone in turn, embedded in epoxy resin, sliced, and stained by way of a saturated uranium acetate and lead citrate double staining method. We then observed and photographed the tissue under an H-7650 transmission electron microscope (Hitachi, Tokyo, Japan).

### Measurement of Mitochondrial Membrane Potential (ΔΨm)

We took heart tissue, embedded it with optimum cutting temperature compound, cut it into 5-μM slices with a cryostat, and placed it on a glass slide to make frozen slices. We pre-incubated it with 5 μmol/L of JC-1 for 10 min at 37°C and washed it twice with 4-(2-hydroxyethyl)-1-piperazineethanesulfonic acid buffer without JC-1. The section was observed under a fluorescence microscope.

### Adenosine Triphosphate Content Determination

The myocardial tissue was lysed with ATP-detection lysate. According to the need for ATP to provide energy when luciferase catalyzes luciferin, the ATP content was measured with a detection kit (S0026B; Biyuntian, Shanghai, China).

### Apoptosis Analysis

The heart tissue sections were incubated for 1 h at 60°C, washed in xylene, and rehydrated through a graded series of ethanol and double distilled water. Samples were rinsed with phosphate-buffered saline 3 times for 5 min each and digested with fresh proteinase K at 37°C for 15–30 min. The tissue sections were then incubated with TUNEL (annexin V-Alexa Fluor 488, 40405ES03) at 37°C for 60 min in the dark, rinsed with phosphate-buffered saline 3 times for 5 min each, and stained with DAB for 5–10 min. The sections were subsequently stained with hematoxylin, washed, dehydrated, mounted, and sealed with neutral gum. Samples were analyzed under a fluorescence microscope at 515–565 nm (green). Levels of myocardial apoptosis were observed by fluorescence microscopy (BX43F-R; Olympus Corporation, Tokyo, Japan).

### Western Blot Analysis

Proteins were extracted from heart tissue, respectively. Electrophoresis was carried out with 12% sodium dodecyl sulfate–polyacrylamide gel electrophoresis gel; then, 25 v of constant pressure was applied to the polyvinylidene fluoride membrane (MilliporeSigma, Burlington, MA, United States) for 30 min, 8% defatted milk powder was sealed at room temperature for 2 h, and corresponding primary antibodies were added at 4°C overnight. The primary antibodies included polyclonal anti-Bax (1:1,000; Abcam, Cambridge, UK), polyclonal anti–Bcl-2 (1:1,000; Abcam, Cambridge, UK), polyclonal anti–caspase-3 (1:1,000; Abcam, Cambridge, UK), polyclonal anti-RyR2 (1:1,000; Abcam, Cambridge, UK), polyclonal anti-PLB (1:1,000; Abcam, Cambridge, UK), polyclonal anti–RyR2-S2808 (1:1,000; Abcam, Cambridge, UK), polyclonal anti–PLB-S16 (1:1,000; Abcam, Cambridge, UK), and glyceraldehyde-3-phosphate dehydrogenase (1:1,000; MDL Biotech Co., Ltd., Beijing, China). After the membrane was washed, the blots were incubated with horseradish peroxidase–conjugated secondary antibodies (1:10,000; Abcam, Cambridge, UK) for 1 h at room temperature, and enhanced chemiluminescence (Vilber Lourmat, France) was carried out after washing the membrane again. The gray values of corresponding protein bands were analyzed and compared to the gray values of internal reference bands. The ratio was the relative expression of proteins.

### Statistical Analysis

For *in vitro* studies, all experiments were performed in triplicate. The data are expressed as mean ± standard error. Quantitative data were tested for normality using the Shapiro–Wilk test, and normally distributed data were evaluated by 1-way analysis of variance using the SPSS version 19.0 software (IBM Corporation, Armonk, NY, United States). In all cases, *p* < 0.05 was considered to be statistically significant.

### Ethics Statement

All experimental protocols were approved by the Experimental Animal Ethics Committee of Hebei University of Chinese Medicine (protocol code, 2016102; date of approval, 25 November 2016). All institutional and national guidelines for the care and use of laboratory animals were followed.

## Results

### Effect of Rhy on Blood Glucose

Before the experiment, the blood glucose concentrations of all mice were detected. Those with fasting blood glucose levels of < 5.6 mmol/L were selected for the experiment, and 10 mice were randomly selected as the blank (control) group (the average blood glucose level was 4.8 mmol/L). The remaining mice were fed a high-fat diet combined with small doses of STZ to replicate the T2DM model, and the successfully modeled mice were divided into three groups evenly according to their blood glucose levels—namely, a T2DM group, a DTL group, and a Rhy group.

**FIGURE 2:**
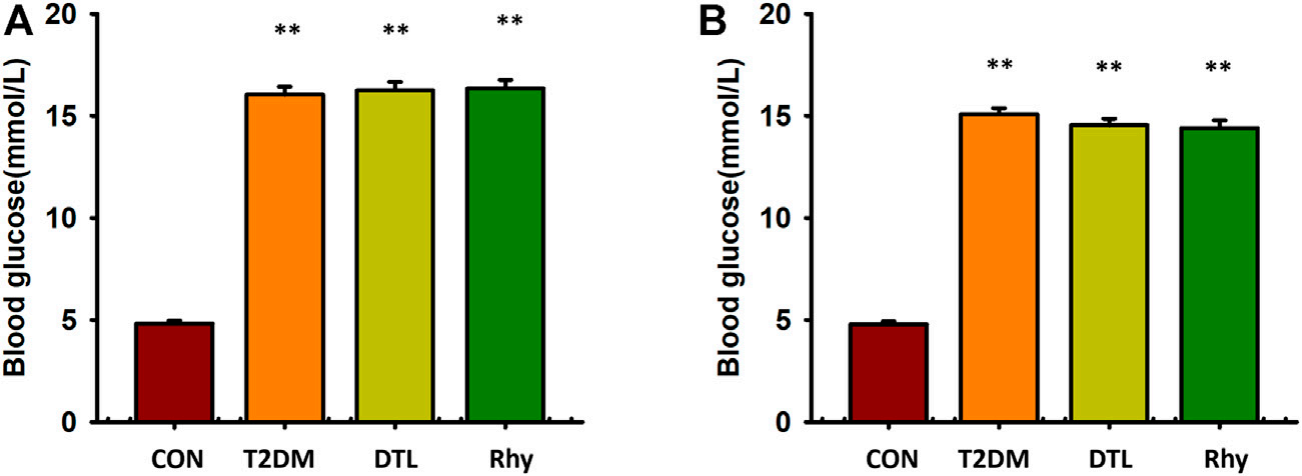
Effect of rhynchophylline on blood glucose. **(A)** The bar graph showed the fasting blood glucose levels of mice in the CON group, T2DM mice, DTL group and Rhy group before administration. **(B)** Blood glucose levels measured 2 months after administration. CON:control group mice; T2DM: Type 2 diabetes model group mice; DTL: T2DM mice treated with Dantrolene (DTL) for 8 weeks; Rhy: T2DM mice treated with rhynchophylline for 8 weeks. Values are presented as mean ± standard error of the mean. **p < 0.01 vs. control group (*n* = 9–10, 1-way analysis of variance, Holm–Sidak post-hoc test).

As shown in Figure 2A, the fasting blood glucose levels of mice in the T2DM group, DTL group, and Rhy group showed no statistical difference between the groups (*p* > 0.05) and were significantly higher than those in the control group (*p* < 0.01). During the 8 weeks of drug intervention, the diabetic group was always in a state of hyperglycemia (Figure 2B), and the fasting blood glucose levels of diabetic mice in the DTL and Rhy groups were significantly higher than those in the control group (*p* < 0.01).

### Effect of Rhy on Cardiac Function

**FIGURE 3:**
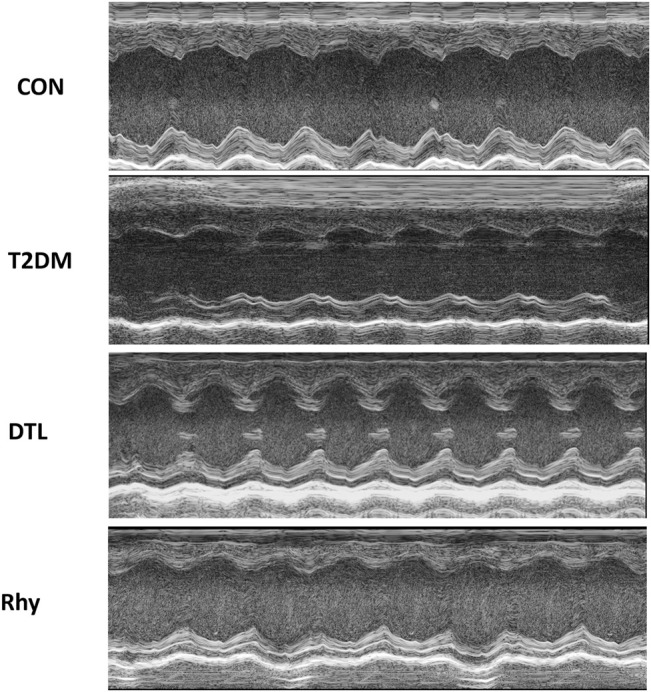
The effects of rhynchophylline on the echocardiographic parameters. Representative M-mode images of left ventricular diameters and wall thicknesses.

Echocardiography (Figure 3) revealed that the heart rates of mice with diabetes for 2 months were significantly higher than those of control mice (*p* < 0.01). Meanwhile, the heart rates of mice in the DTL and Rhy groups were significantly reduced (*p* < 0.01) to nearly normal (Supplementary Table S1).

From the results of echocardiography, the cardiac chamber size was typically smaller in the T2MD group than the control group (Figure 3). The EF, FS, and LVEDV in the T2MD group were significantly reduced (*p* < 0.01 or *p* < 0.05), while the LVESV, IVSd, and IVSs were significantly increased (*p* < 0.01). Additionally, the LVPWd and LVPWs in the T2DM group were not statistically different from those in the control group (*p* > 0.05), but they also had a tendency to increase. The cardiac cavity size of mice treated with DTL or Rhy was increased. There was a significant increase in EF in the Rhy group compared to the T2MD group (*p* < 0.05). Compared to the T2DM group, there was significantly decrease in heart rate in the Rhy group (*p* < 0.01). In the DTL and Rhy groups, LVESV levels were significantly lower (*p* < 0.05 or *p* < 0.01) and some improvements in LVEDV, LVPWd, LVPWs, IVSd, and IVSs were noted (*p* > 0.05).

### Effect of Rhy on Cardiac Appearance and Morphology

**FIGURE 4:**
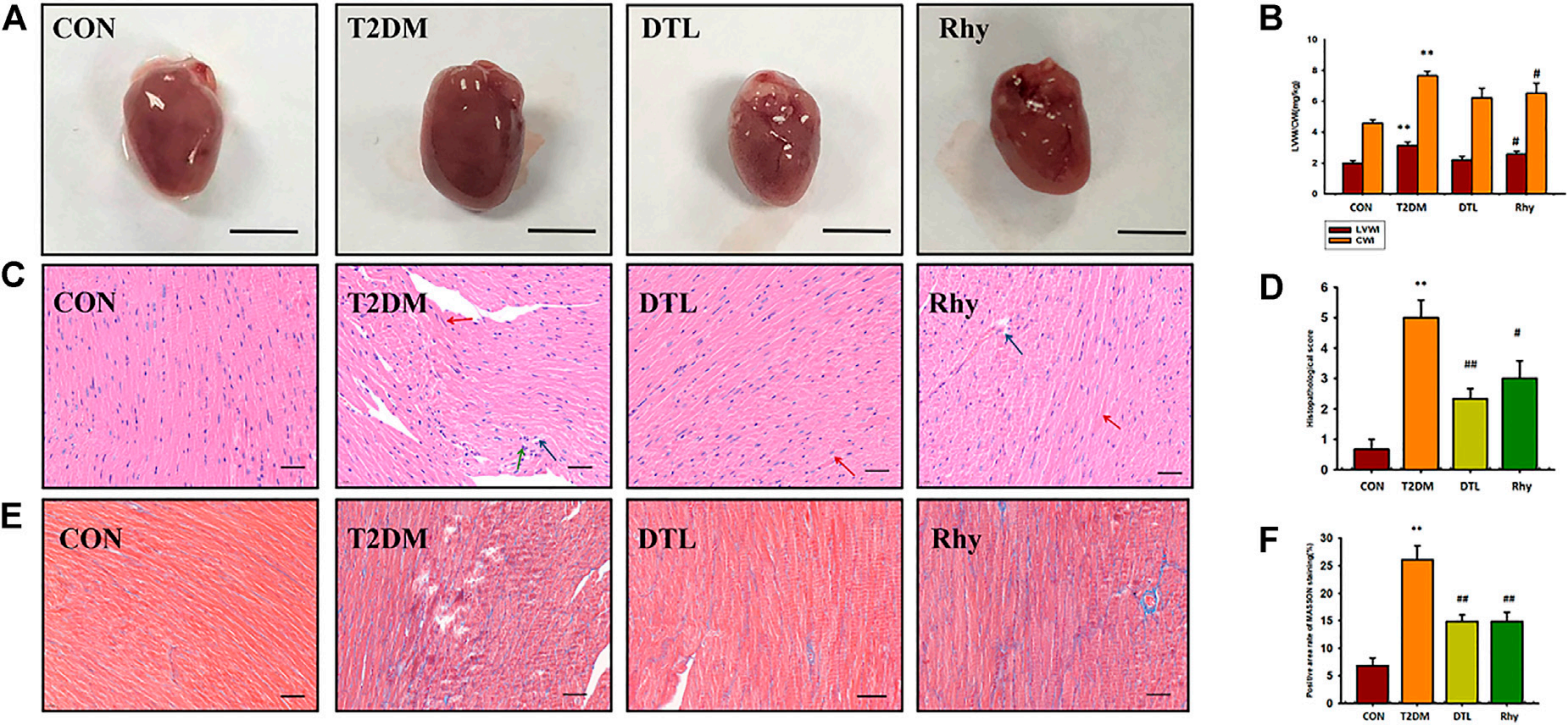
Effect of rhynchophylline on cardiac appearance and morphology. **(A)** Representative heart photographs of each group. **(B)** The left ventricular weight index (LVWI) and cardiac weight index (CWI). **(C)** Representative sections (magnification, ×400) of cardiac tissue morphology stained by hematoxylin and eosin in each group. **(D)** Pathology scores; **(E)** Representative sections (magnification, ×400) of cardiac tissue morphology stained by Masson’s trichrome stain in each group; **(F)** Positive area rate of MASSON staining. Values are presented as mean ± standard error of the mean. **p < 0.01 vs. control group; #*p* < 0.05 vs. type 2 diabetes mellitus group.

Figure 4A shows the appearance of mouse hearts from each group. It can be seen from the figure that the hearts of mice with diabetes for 2 months became swollen and brown, and the LV weight index (LVWI) and cardiac weight index (CWI) were both much higher than normal (*p* < 0.01, Figure 4B). After 8 weeks of treatment with DTL and Rhy, this change was significantly attenuated, making the heart appear close to normal, with a significant reduction in both LVWI and CWI (DTL group, *p* < 0.05).

Myocardial overgrowth and connective tissue hyperplasia play a key role in diabetes-induced myocardial remodeling. Therefore, we used hematoxylin and eosin and Masson’s trichrome staining to assess cardiac histopathological alterations in T2DM mice. Figure 4C shows the pathomorphological changes of heart tissue under a light microscope. It can be seen from Figure 4C that the section of normal mouse myocardium has clear transverse lines, and cell nuclei are in the middle. The myocardial interstitium of T2DM mice was widened (red arrow), with local inflammatory cell infiltration (green arrow), myocardial cell degeneration and necrosis, and lipid droplet vacuoles in the cytoplasm of cardiomyocytes (blue arrow), and the pathology scores of the T2DM group were significantly higher than those of the control group (*p* < 0.01, Figure 4D). The DTL and Rhy groups were similar, and the morphological changes of the heart tissue of mice in either group were greatly improved compared to T2DM mice (*p* < 0.01 or *p* < 0.05, Figures 4D,E) shows the heart tissue morphology stained by Masson’s trichrome stain. We can see that the degree of myocardial interstitial fibrosis in T2DM mice was significantly higher than that in the control group (*p* < 0.01, Figure 4F). After treatment with DTL and Rhy, the degree of myocardial fibrosis in mice was significantly improved compared to in the T2DM group (*p* < 0.01, Figure 4F).

### Effects of Rhy on Cardiac Injury–Associated Enzymes

**FIGURE 5:**
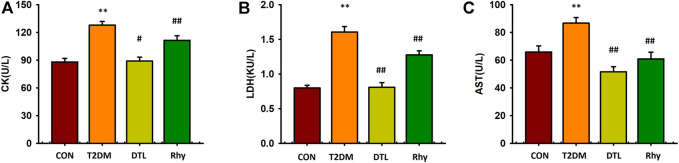
Effect of rhynchophylline on cardiac enzymes. **(A)** The levels of creatine kinase (CK). **(B)** The levels of lactate dehydrogenase (LDH). **(C)** The levels of aspartate aminotransferase (AST). Values are presented as mean ± standard error of the mean. **p < 0.01 vs. control group; #*p* < 0.05, ##*p* < 0.01 vs. type 2 diabetes mellitus group.

Cardiac enzymes, such as CK, LDH, and AST, are mainly found in cardiac myocytes. However, myocardial enzymes can be released into the serum due to enhanced myocardial cell permeability during myocardial ischemia and acute myocardial infarction. Therefore, the measurement of cardiac enzymes in serum is valuable for the diagnosis of myocardial cell injury. CK, LDH, and AST levels in mice with diabetes for 2 months were significantly higher than those in the control group (*p* < 0.01, Figure 5). Compared to T2MD mice, the levels of these three cardiac enzymes in the serum of DTL and Rhy mice were significantly lower (*p* < 0.01 or *p* < 0.05).

### Effect of Rhy on the Degree of Apoptosis in Heart Tissue

**FIGURE 6:**
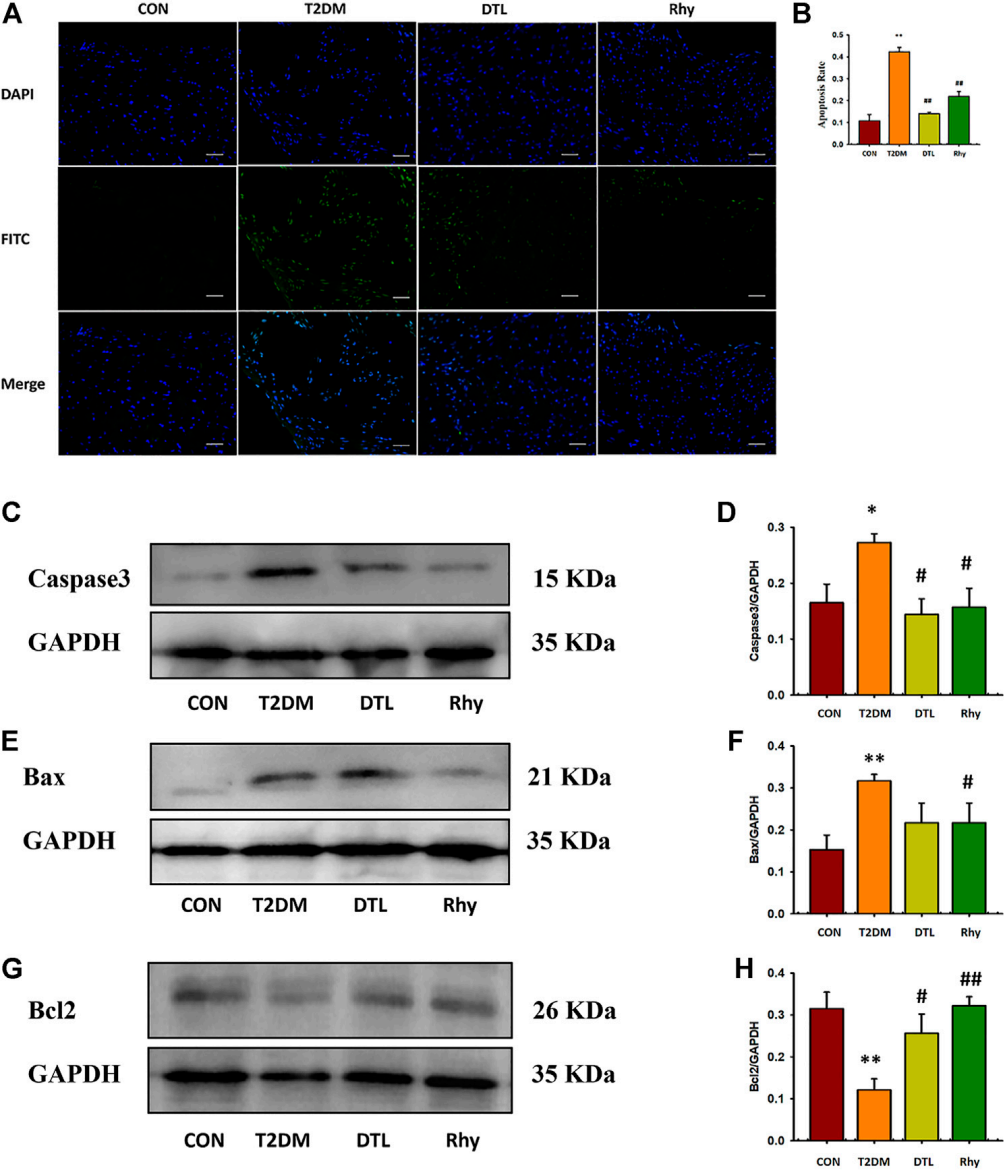
Effect of rhynchophylline on the degree of apoptosis in cardiac tissue. **(A)** Representative sections of apoptosis in cardiac tissues of each group (magnification, ×200). **(B)** The degree of apoptosis in cardiac tissue. **(C–H)** Caspase-3, Bax, and Bcl-2 protein expressions in heart tissue were determined by western blot analysis. Relative intensities of caspase-3 **(D)**, Bax **(F)**, and Bcl-2 **(H)** were calculated by normalization to glyceraldehyde-3-phosphate dehydrogenase in each group; Values are presented as mean ± standard error of the mean. *p < 0.05, **p < 0.01 vs. control group; #*p* < 0.05, ##*p* < 0.01 vs. type 2 diabetes mellitus group (*n* = 3, 1-way analysis of variance, Holm–Sidak post-hoc test).

In this study, we observed the apoptosis of myocardial cells and the expression level of apoptosis protein. As reflected in Figure 6, the degree of apoptosis in heart tissues of T2DM mice was significantly higher than that of normal mice (*p* < 0.01, Figures 6A,B), and the expressions of apoptosis proteins caspase-3 (Figures 6C,D) and Bax (Figures 6E,F) in tissues were significantly upregulated (*p* < 0.01 or *p* < 0.05), while the expression of Bcl-2 protein was significantly decreased (*p* < 0.01, Figures 6G,H). Following intervention with DTL and Rhy, the apoptosis level of tissue cells decreased (*p* < 0.01), and the expression levels of caspase-3 (*p* < 0.05) and Bax (Rhy group, *p* < 0.05) protein were reduced to some extent, while the expression of Bcl-2 protein was increased significantly (*p* < 0.01 or *p* < 0.05).

### Effect of Rhy on Myocardial Contraction

**FIGURE 7:**
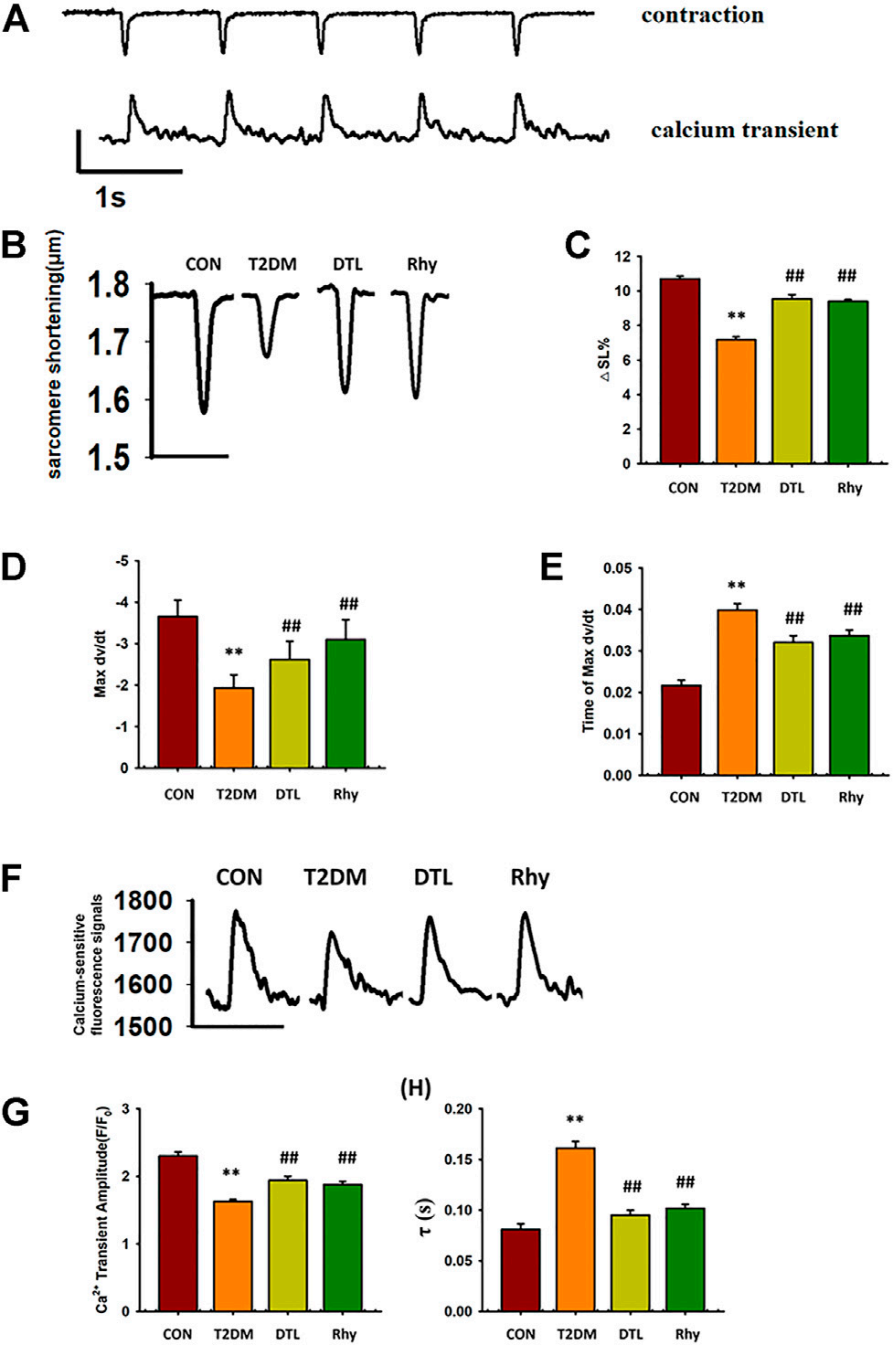
Effect of rhynchophylline on cardiomyocyte contraction and calcium transient. **(A)** Representative real-time monitoring of ventricular myocyte contraction and calcium transience images. **(B)** Representative monitoring of ventricular myocyte contraction in each group. **(C)** The amplitude of cardiomyocyte contraction. **(D)** The maximum rate of cell contraction. **(E)** The time for cells to reach the maximum rate of contraction. **(F)** Representative monitoring of ventricular myocyte calcium transience in each group. **(G)** The magnitude of calcium transience (△F/F_0_). **(H)** The tau value(τ) of calcium transience. Values are presented as mean ± standard error of the mean. **p < 0.01 vs. control group; #*p* < 0.05, ##*p* < 0.01 vs. type 2 diabetes mellitus group. *Abbreviations:* N, mouse; n, cell. *n* = 3 each group, n ≥ 10 each group, 1-way analysis of variance, Holm–Sidak post-hoc test.

In the experiment, we simultaneously examined the dynamic changes in contraction amplitude and cytoplasmic calcium ion concentration in ventricular myocytes (Figures 7A,B) shows representative traces depicting the typical effects of DTL and Rhy on cell shortening in cardiomyocytes. Compared to control group mice, the amplitude of cardiomyocyte contraction in T2DM mice was significantly reduced (*p* < 0.01, Figure 7C), the maximum rate of cell contraction was significantly reduced (*p* < 0.01, Figure 7D), and the time for cells to reach the maximum rate of contraction was significantly prolonged (*p* < 0.01, Figure 7E). After treatment with DTL and Rhy, the contraction amplitude of mouse cardiomyocytes was significantly increased (*p* < 0.01), the maximum rate of cell contraction was significantly accelerated (*p* < 0.01), and the time to reach the maximum rate of cell contraction was significantly shortened (*p* < 0.01).

### Effect of Rhy on Calcium Transience in Cardiomyocytes

In mouse ventricular myocytes, we detected the level of free Ca^2+^ in cytoplasm by loading Fluo-4 AM. Figure 7F shows the typical changes in calcium ion concentration in cytoplasm accompanied by the excitation–contraction coupling of mouse cardiomyocytes in each group. From the results of Figures 7G,H, we can see that, compared to normal mice, the magnitude of calcium transience in mice with diabetes for 2 months was decreased significantly (*p* < 0.01) and the tau value was increased significantly (*p* < 0.01). However, the parameters of these calcium transients were significantly improved in both DTL- and Rhy-treated mice (*p* < 0.01). Comparing the basal calcium-sensitive fluorescent signals in the cytoplasm of cardiomyocytes in each group, we found that those in the T2DM group were significantly higher than normal (*p* < 0.01), while treatment with DTL or Rhy reduced the signal intensity (*p* < 0.05, Supplementary Figure S1). It was found that SR spontaneous Ca^2+^ release was increased in cardiomyocytes of T2DM mice due to excessive activation of RyR2, while both DTL and Rhy treatment reduced the number of releases (Supplementary Figure S2). This result indicated that Rhy inhibits excessive RyR2 activity and can effectively reduce SR calcium leakage.

### Effect of Rhy on the Phosphorylation Level of RyR2 and PLB Protein in Cardiomyocytes

**FIGURE 8:**
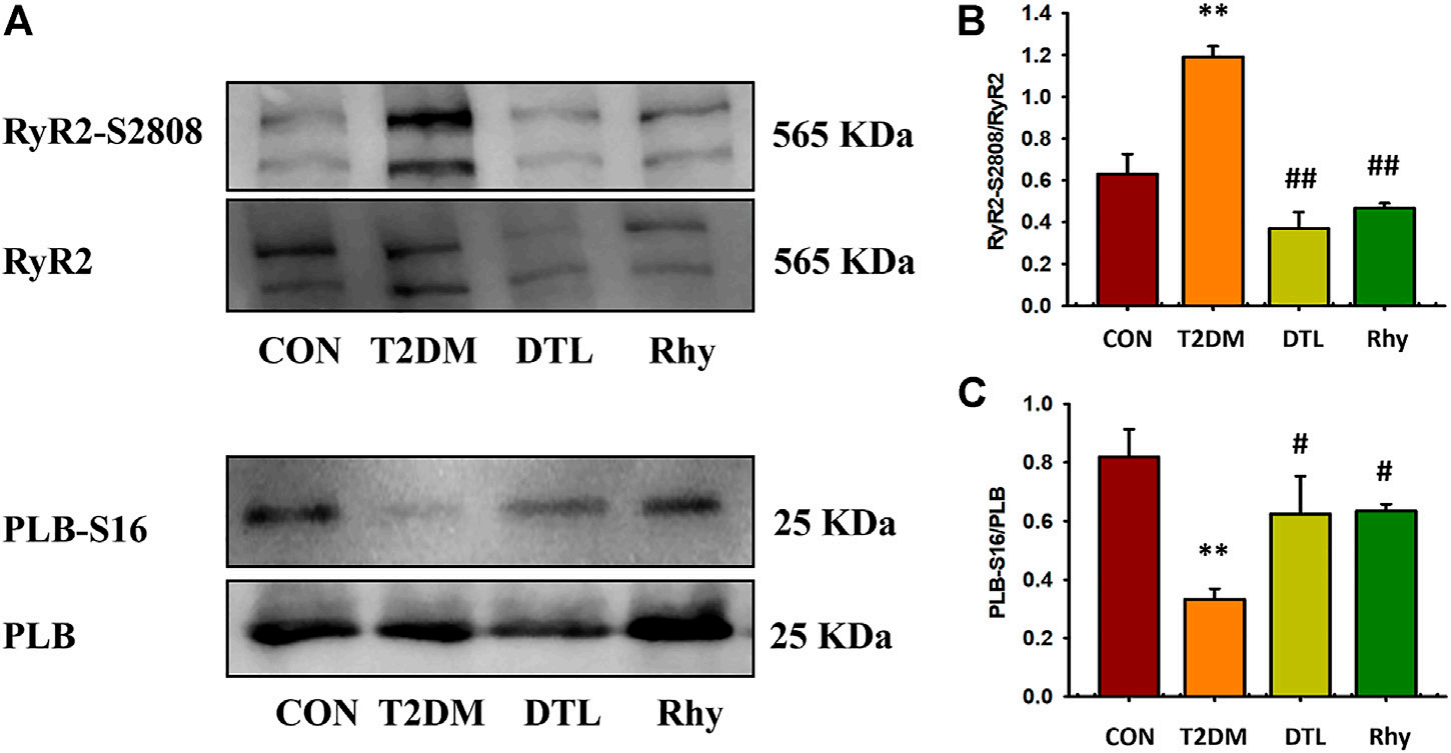
Effect of rhynchophylline on the phosphorylation levels of ryanodine receptor 2 (RyR2) and PLB protein in cardiomyocytes. **(A)** The phosphorylation protein-expression levels of RyR2-S2808 and PLB-S16 were detected by Western blot analysis. **(B)** The phosphorylation levels of RyR2. **(C)** The phosphorylation levels of PLB. Values are presented as mean ± standard error of the mean. **p < 0.01 vs. control group; #*p* < 0.05, ##*p* < 0.01 vs. type 2 diabetes mellitus group.

The amplitude and time course of myocardial calcium transient are regulated by three kinds of proteins—namely, L-shaped calcium channels on the cell membrane, sarcoplasmic omentum RyR2, and calcium ATPase (calcium pump) (Lou et al., 2012). Al Kury LT reported that STZ-induced diabetes resulted in an change in amplitude of Ca^2+^ transients in myocytes that was independent of the LTCC current. So we mainly observed the effect of Rhy on the activities of RyR2 and SERCA in the present study. The binding of SERCA and Ca^2+^ is influenced by the phosphorylation level of PLB, so we evaluated the activity of SERCA by observing the intervention of Rhy on this index. According to the results shown in Figure 8A, compared to the control group, the phosphorylation level of RyR2 (RyR2-S2802/RyR2) in the T2MD group was significantly increased (*p* < 0.05, Figure 8B), while that of PLB (PLB-S16/PLB) was significantly decreased (*p* < 0.01, Figure 8C). After DTL or Rhy treatment, these parameters were significantly recovered (*p* < 0.01 or *p* < 0.05).

### Effect of Rhy on the Microstructure and Mitochondria Morphology of Cardiomyocytes

**FIGURE 9:**
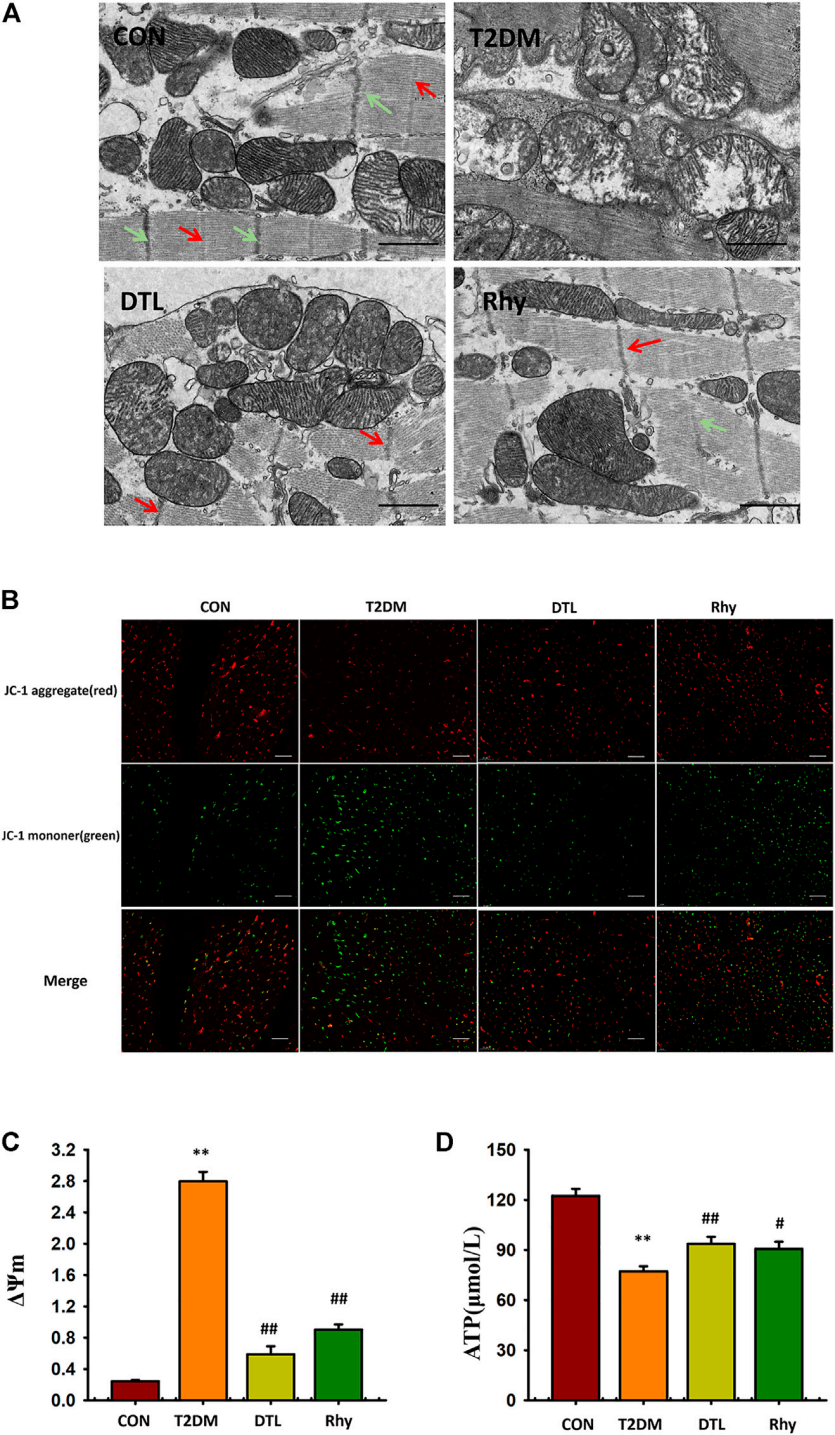
Effect of rhynchophylline on the microstructure and mitochondria morphology of cardiomyocytes. **(A)** Representative sections of mouse cardiomyocytes observed under a transmission electron microscope. **(B)** Representative mitochondrial membrane potential observed under a fluorescence microscope (magnification, ×200). **(C)** The dynamic change in ΔΨm represented by the ratio of fluorescence intensity of 527 and 590 nm. **(D)** Adenosine triphosphate content in myocardial tissue. Values are presented as mean ± standard error of the mean. **p < 0.01 vs. control group; #*p* < 0.05, ##*p* < 0.01 vs. type 2 diabetes mellitus group.

Figure 9A shows the microstructure of mouse cardiomyocytes observed under a transmission electron microscope. The myocardial myofibrils of normal mice are connected to each other, arranged neatly and closely; the M line (red arrow) and Z line (green arrow) are clearly visible, the mitochondria are abundant, and their structure is clear, with no obvious vacuoles inside and an intact envelope and cristae. In contrast, cardiomyocytes from the T2DM group showed disordered fiber arrangement, M line and Z line breakage, mitochondrial swelling and degeneration, and vacuoles. After the intervention of drugs, the microstructure of cells and the morphology of mitochondria were obviously improved.

### Effect of Rhy on Mitochondrial Membrane Potential in Cardiac Tissue

In this study, we used JC-1–loaded myocardial tissue as a response to carbonyl cyanide 4- (trifluoromethoxy) phenylhydrazone to measure mitochondrial membrane potential. The excitation wavelength was 527 nm, showing green fluorescence, which indicated that the membrane potential level was low; the excitation wavelength was 590 nm, showing red fluorescence, which indicated that the membrane potential level was high. The results were expressed by the dynamic change in ΔΨm represented by the ratio of fluorescence intensity of 527 and 590 nm. As shown in Figures 9B,C, compared to the control group, ΔΨm in the T2DM group was increased (*p* < 0.01). The ΔΨm of cardiac tissue in mice treated with DTL and Rhy was significantly lower than that in the T2DM group (*p* < 0.01).

### Effect of Rhy on Adenosine Triphosphate Content in Heart Tissue

As shown in Figure 9D, compared to the control group, the ATP level in myocardium of T2DM group mice was significantly lower (*p* < 0.01). After DTL or Rhy treatment, the content of ATP in tissues increased significantly (*p* < 0.01 or *p* < 0.05).

## Discussion

In the process of diabetes onset, chronic hyperglycemia will produce a variety of complications, including lesions of microvessels, macrovessels, and the myocardium. At present, the correlation between abnormal cardiac function and diabetes has been well confirmed (Jia et al., 2018). The treatment of DCM includes medications or surgery but remains a challenge. The findings of the present study showed that Rhy could ameliorate myocardial injury in T2DM mice, and its effect might be correlated with the regulation of intracellular calcium homeostasis in cardiac myocytes.

In this study, we replicated a T2DM mouse model using a high-fat diet combined with a small amount of multiple injections of STZ (Deng et al., 2020). At 2 months of illness, the fasting blood glucose levels of diabetic mice were significantly higher than those of controls. Then, we detected the cardiac function of mice using small-animal B-ultrasound imaging, checked the myocardial enzyme levels of myocardial tissue, and made pathological observations of the cardiac tissue. Similar to the experimental results reported in the literature (Chen et al., 2022), T2DM mice exhibited myocardial hypertrophy, increased heart rate, reduced LV systolic and diastolic function, reduced EF, and abnormally high serum levels of cardiac enzymes. These results confirmed that diabetes can cause myocardial damage. Echocardiographic assessment of T2DM mice in our study was similar to that of clinical DCM patients, indicating a successful model replication.

When Rhy was used to treat T2DM mice, although the blood glucose did not return to the its normal level, Rhy had a good protective effect on myocardial damage caused by diabetes. This result suggested that the ameliorating effect of Rhy on diabetic cardiomyopathy is not achieved by lowering the blood glucose. In this manuscript, we explored the mechanism of how Rhy regulates myocardial contraction.

The contraction of the myocardium is composed of the excited contraction of cardiac muscle cells, and the increase and decrease in intracellular Ca^2+^ concentrations is key to the contraction of cardiomyocytes (Njegic et al., 2020). Therefore, we observed the effects of Rhy on cardiomyocyte contraction and intracellular calcium transient. The experimental results showed that, after Rhy treatment, the contraction amplitude of the previously inhibited cardiomyocytes in T2DM mice increased, the cytosolic Ca^2+^ levels during diastole decreased, the level of intracellular calcium transient increased, and the calcium recovery rate of the sarcoplasmic reticulum was greatly improved. Many studies have reported no change in LTCC currents in ventricular cells from STZ-treated rat (Shao et al., 2007; Al Kury et al., 2021). Therefore, our manuscript focused on the effect of Rhy on sarcoplasmic reticulum calcium release. According to existing literature reports, DTL is a stabilizer of RyR2 that could protect cardiomyocytes by regulating intracellular calcium homeostasis (Azam et al., 2021). Therefore, this chemical was chosen as a positive control drug in this study.

Abnormal activity of RyR2 and calcium leakage from the sarcoplasmic reticulum have been reported to cause myocardial injury and cardiac remodeling. In this study, we found that, compared to normal mice, the phosphorylation level of RyR2 in cardiomyocytes of T2DM mice was higher, while that of PLB was lower. The hyperphosphorylation of RyR2 increases its opening probability, which increases the calcium leakage in the sarcoplasmic reticulum, decreases the calcium pump reserve, and inhibits myocardial cell contraction (Ruiz-Meana et al., 2019). It is a manifestation of calcium leakage that we observed in T2DM mice with increased spontaneous calcium release from the sarcoplasmic reticulum of ventricular myocytes. However, following treatment with Rhy, similar to the effect of DTL, the phosphorylation level of RyR2 decreased, while the phosphorylation level of PLB tended to increase, reducing calcium leakage and promoting calcium recovery, which may be one of the causes behind the reduction in sarcoplasmic reticulum calcium overload.

Whenthere was calcium overload in the cytoplasm of T2DM mice, the mitochondria absorbed Ca^2+^ rapidly, causing an accumulation of Ca^2+^ in the mitochondria and then mitochondrial damage (Wu et al., 2019). Thus, we could observe by electron microscopy that the mitochondrial membrane structure was damaged and cristae breaks were created. When the mitochondrial structure is damaged, the mitochondrial membrane potential decreases and the ATP level of the heart also decreases (Contreras-Ferrat et al., 2014). Due to the reduced cytosolic calcium ion concentration, Rhy protected the structure and function of mitochondria and maintained the level of cardiac ATP at a point significantly higher than that in T2DM mice. ATP content is one of the factors affecting SERCA activity, and the increase is ATP level may also be one of the factors behind sarcoplasmic reticulum calcium recovery (Sakamoto & Tonomura, 1980).

Both Bax and Bcl2 are closely related to the mitochondrial apoptotic response (Lam et al., 1994). Bcl-2 and Bax are able to insert into the mitochondrial membrane, forming ion channels and changing the membrane permeability (Danial & Korsmeyer, 2004). By regulating Bax and Bcl-2, caspase-3, the executor of apoptosis, can hydrolyze various cell components and cause cell apoptosis. However, in this experiment, we found that the expressions of Bax and caspase-3 in the heart tissue of T2DM mice were significantly upregulated, the expression of Bcl-2 was downregulated, and the rate of apoptosis was significantly increased. Rhy could protect cardiomyocytes and reduce apoptosis.

**FIGURE 10:**
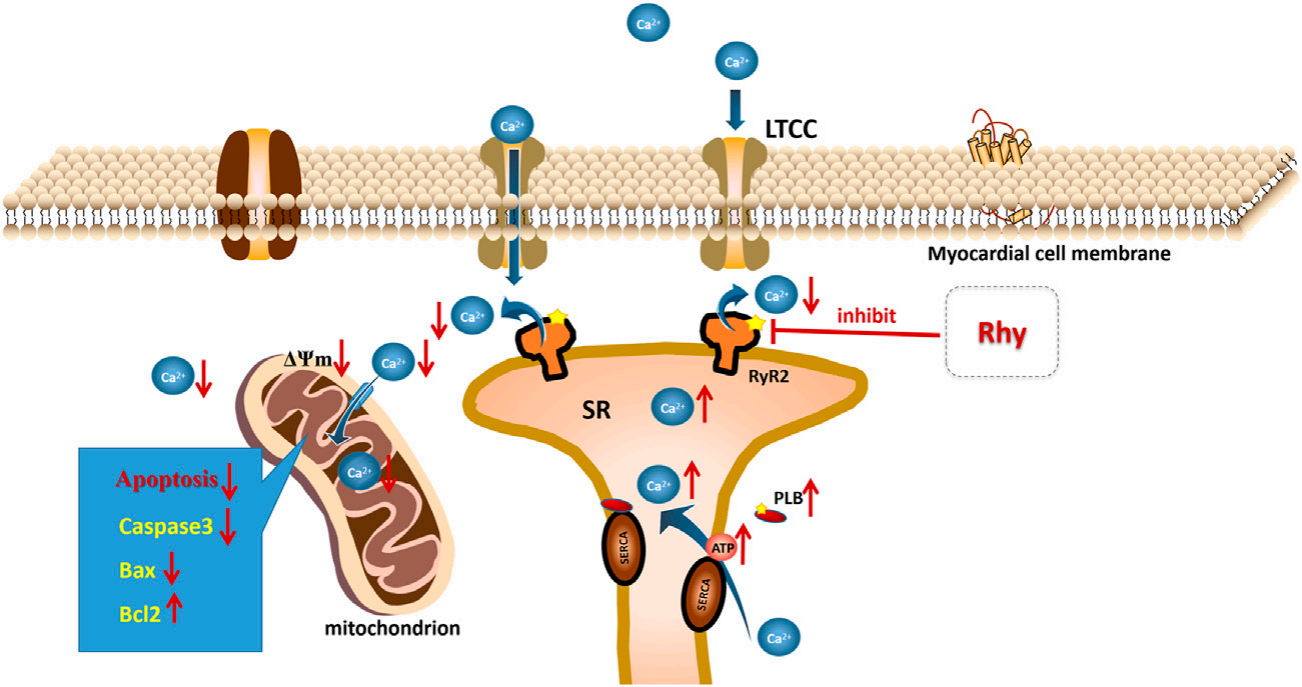
Mechanism of rhynchophylline to improve diabetic cardiomyopathy.

Our study found that Rhy, similar to DTL, had a stronger effect on RyR2 activity than on PLB and therefore had a significant promotional impact on ventricular contraction. Based on this finding, we hypothesized that RyR2 may be the target of Rhy action and that the elevated PLB phosphorylation level may be indirectly caused by reduced calcium leakage from the sarcoplasmic reticulum, restored mitochondrial function, and elevated ATP levels (Figure 10). However, our description of this process is still only speculation, and we will continue to investigate the endocalcium regulatory role of Rhy in order to clarify its direct targets in future studies.

## Conclusion

In a word, our results have demonstrated that Rhy can protect against myocardial damage in T2DM mice and promote cardiomyocyte contraction, and its mechanism of action is related to the regulation of intracellular calcium homeostasis. Rhy may be able to inhibit the overactivation of sarcoplasmic reticulum RyR2 and reduce calcium leakage and calcium overload in cytoplasm, thereby decreasing the concentration of Ca^2+^ in mitochondria, protecting the structure and function of mitochondria, increasing ATP levels in heart tissue, enhancing the activity of SERCA, increasing calcium recovery in the sarcoplasmic reticulum, and further reducing calcium overload. The results suggest that the application of Rhy may provide a new strategy for the prevention of diabetic cardiomyopathy.

## Data Availability

The original contributions presented in the study are included in the article/Supplementary Material, further inquiries can be directed to the corresponding authors.
