# Supplementary figures and images for: Rhynchophylline Regulates Calcium Homeostasis by Antagonizing Ryanodine Receptor 2 Phosphorylation to Improve Diabetic Cardiomyopathy
Source: Front Pharmacol. 2022 Apr 19;13:882198. doi: 10.3389/fphar.2022.882198 (PMC9063879; doi:10.3389/fphar.2022.882198)

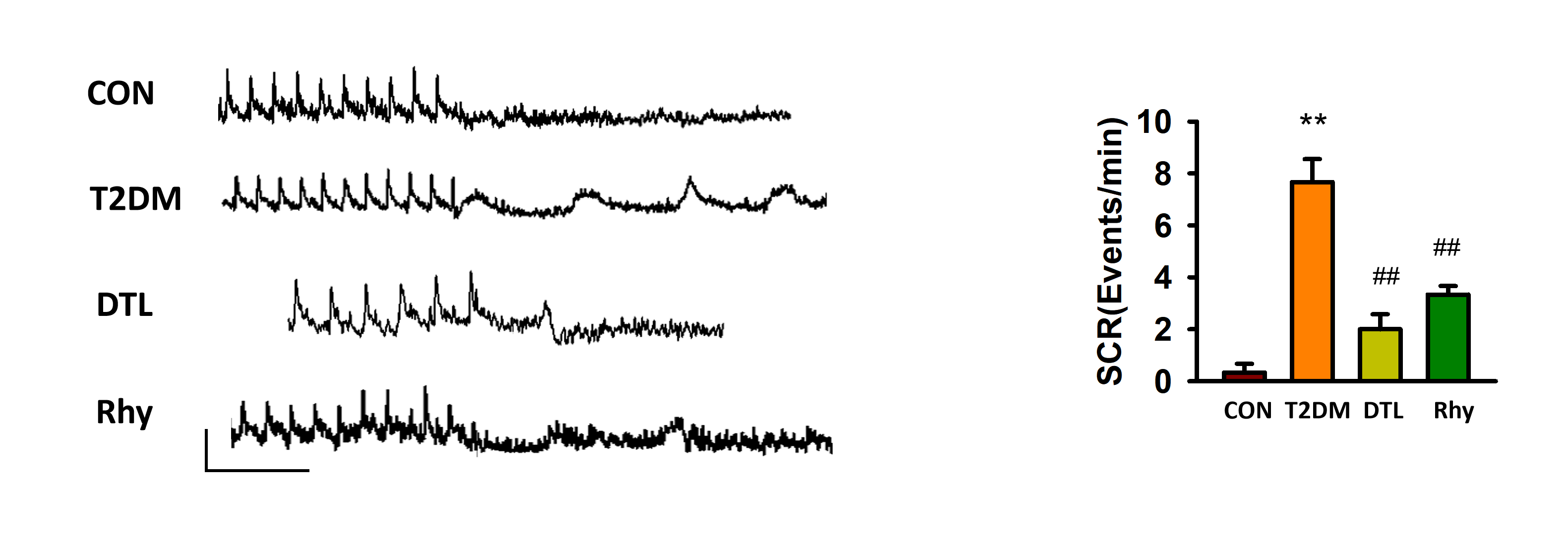

Supplement: Supplementary file 1 [file Image2.TIF]

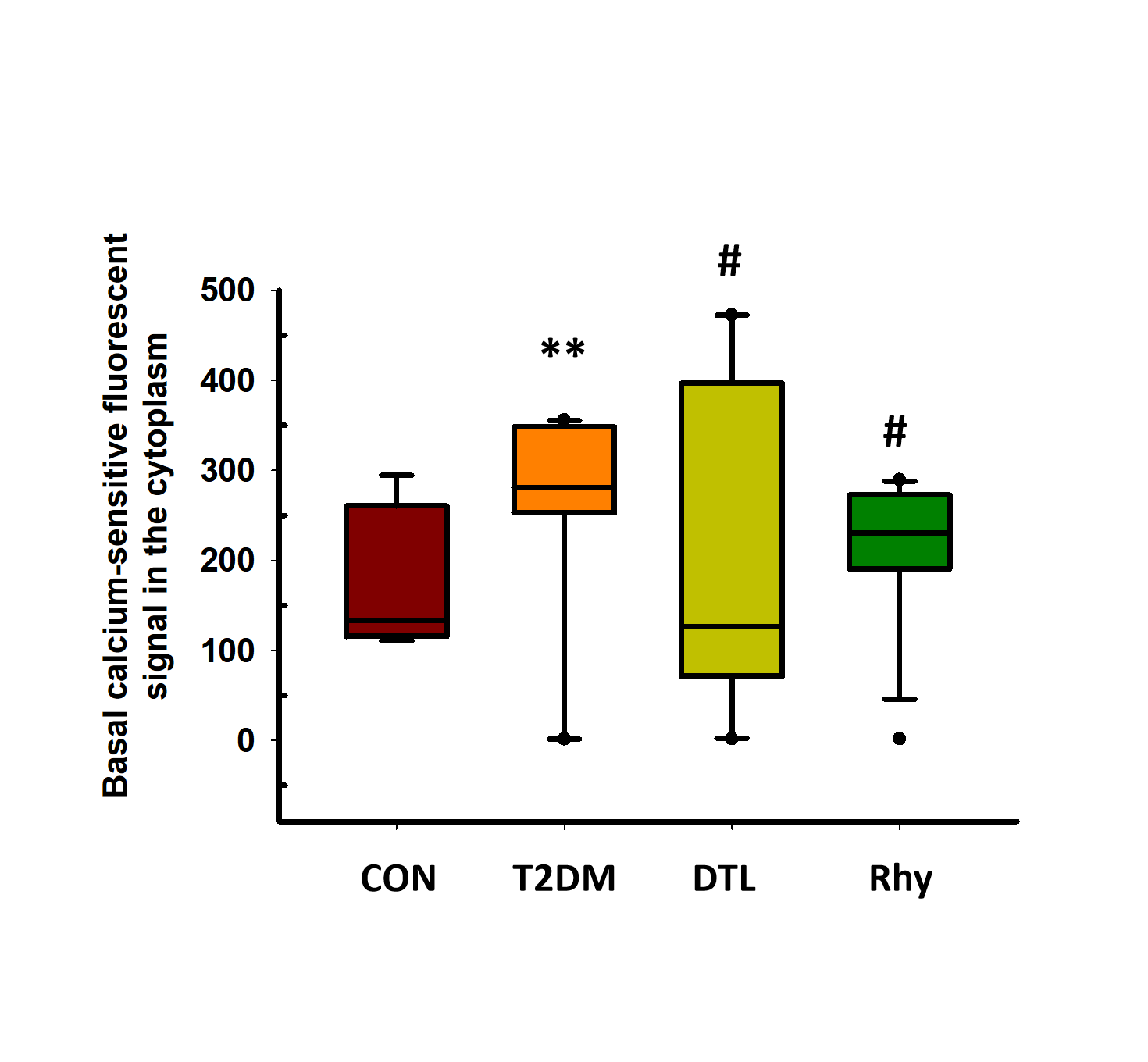

Supplement: Supplementary file 2 [file Image1.TIF]
