# Supplementary material for: Rhynchophylline Regulates Calcium Homeostasis by Antagonizing Ryanodine Receptor 2 Phosphorylation to Improve Diabetic Cardiomyopathy
Source: Front Pharmacol. 2022 Apr 19;13:882198. doi: 10.3389/fphar.2022.882198 (PMC9063879; doi:10.3389/fphar.2022.882198)
Supplement: Supplementary file 3 [file Table1.DOC]

**Table 1 M-mode Echocardiography in Small Animals to**

**Detect Left ventricular (LV) Function**

| **Testing index** | **CON** | **T2DM** | **DTL** | **Rhy** |
| --- | --- | --- | --- | --- |
| **Heart Rate(beats/min)** | 376.0±16.2 | 507.8±15.2^**^ | 415.0±13.5^##^ | 349±29.4^##^ |
| **Ejection Fraction(%)** | 60.9±2.9 | 45.4±1.7^**^ | 52.5±2.3 | 57.1±4.5^#^ |
| **Fraction Shortening(%)** | 32.7±2.7 | 24.1±1.7^*^ | 26.5±1.4 | 30.9±3.1 |
| **LVESV(μL)** | 26.4±2.6 | 37.5±2.4^**^ | 28.9±1.7^#^ | 26.4±1.7^##^ |
| **LVEDV(μL)** | 77.5±3.0 | 59.3±4.8^**^ | 61.0±3.5 | 64.4±3.0 |
| **LVPWd(mm)** | 0.66±0.08 | 0.82±0.06 | 0.73±0.07 | 0.77±0.08 |
| **LVPWs(mm)** | 1.03±0.06 | 1.20±0.07 | 1.08±0.07 | 1.14±0.14 |
| **IVSd(mm)** | 0.60±0.04 | 0.83±0.06^**^ | 0.76±0.04 | 0.73±0.08 |
| **IVSs(mm)** | 0.67±0.06 | 1.15±0.06^**^ | 0.96±0.07 | 1.03±0.09 |

Values are presentedas mean±SEM; **P*<0.05, ***P*<0.01 vs. control group; #*P*<0.05, ##*P*<0.01 vs. T2DM group.
